# Supplementary material for: Sensitive Detection of Trace Explosives by a Self-Assembled Monolayer Sensor
Source: Micromachines (Basel). 2023 Nov 29;14(12):2179. doi: 10.3390/mi14122179 (PMC10745381; doi:10.3390/mi14122179)
Supplement: Supplementary file 1 [file micromachines-14-02179-s001.zip › micromachines-2727469-supplementary.pdf]

micromachines

Supplementary Information:

Sensitive Detection of Trace Explosives by a Self-Assembled Monolayer Sensor

Weitao Liu [1,†], Wajid Ali [1,†], Ye Liu [1], Mingliang Li [2,*] and Ziwei Li [1,*]

[1] Hunan Institute of Optoelectronic Integration, College of Materials Science and Engineering, Hunan University, Changsha 410082, China; zczqwli@ucl.ac.uk (W.L.); wajidali@hnu.edu.cn (W.A.); liuye455@hnu.edu.cn (Y.L.)
[2] Department of Chemistry, The University of Hong Kong, Hong Kong 999077, China
* Correspondence: liml@hku.hk (M.L.); ziwei_li@hnu.edu.cn (Z.L.)
† These authors contributed equally to this work.

S1: Sensing Tests and Synthesis of SAMs

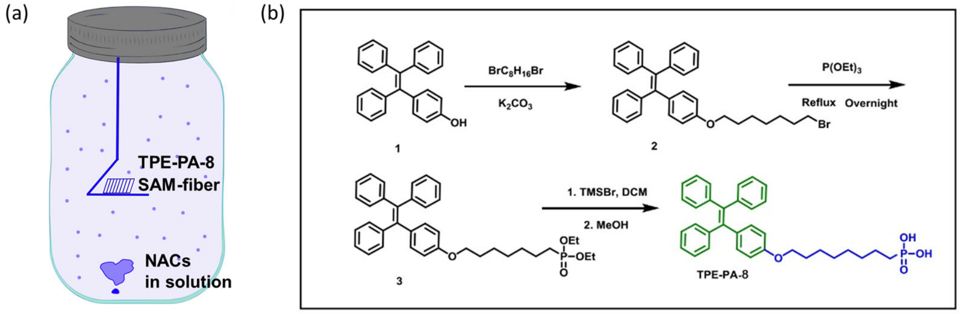

Figure S1. **a** shows the schematic view of sensing tests in a closed container. For sensing tests of NACs, the experiments were conducted by hanging designed SAM-coated fiber films in a closed container, where NACs have been dissolved in methanol solutions and located at the bottom of the container. The solution is left undisturbed for 15 minutes to allow for complete evaporation within the bottle. For one-time sensing test, after the TPE-PA-8 SAM-coated fiber film was positioned in the bottle using a bracket for five minutes, the fiber film was then picked out for fluorescence spectrum detection. **b** shows synthesis process of TPE-PA-8 SAMs.
